# Attention-deficit hyperactivity disorder in school-age children in Gaborone, Botswana: Comorbidity and risk factors

**DOI:** 10.4102/sajpsychiatry.v26i0.1525

**Published:** 2020-10-22

**Authors:** Anthony A. Olashore, Saeeda Paruk, John A. Ogunjumo, Radiance M. Ogundipe

**Affiliations:** 1 Department of Psychiatry, Faculty of Medicine, University of Botswana, Gaborone, Botswana; 2 Department of Psychiatry, Nelson R. Mandela School of Medicine, University of KwaZulu-Natal, Durban South Africa; 3 Department of Family Medicine, Faculty of Medicine, University of Botswana, Gaborone, Botswana

**Keywords:** ADHD, Botswana, children, comorbidity, risk factors

## Abstract

**Background:**

Attention deficit hyperactivity disorder (ADHD) is one of the most common neurodevelopmental disorders in children. Its occurrence and pattern of presentation are unknown in Botswana.

**Aim:**

To determine the prevalence of attention-deficit hyperactivity disorder (ADHD), associated comorbid conditions and risk factors amongst school-age children in Botswana.

**Setting:**

Primary schools in Gaborone, Botswana.

**Methods:**

This study used a cross-sectional design. A two-stage random sampling technique was utilised to select learners from 25 out of the 29 public schools in the city. The Vanderbilt ADHD Diagnostic Rating Scale (VADRS), teacher and parent versions, was administered.

**Results:**

Of the 1737 children, 50.9% (*n* = 884) were male, and their mean age was 9.53 years (s.d. = 1.97). The prevalence of ADHD was 12.3% (*n* = 213). The most prevalent presentation was the predominantly inattentive, 7.2% (*n* = 125). A family history of mental illness (odds ratio [OR] = 6.59, 95% CI: 1.36–32.0) and perinatal complications (OR = 2.16, 95% CI: 1.08–4.29) emerged as the independent predictors of ADHD.

**Conclusions:**

The prevalence of ADHD in Botswana is slightly higher than that reported in the literature, but the pattern of presentations and comorbidities is similar. A positive family history of mental illness and perinatal complications independently predicted ADHD. Mental health screening amongst families of the affected individuals and improved perinatal care should be considered as health care priorities in Botswana.

## Introduction

Attention-deficit hyperactivity disorder (ADHD) is one of the most common neurodevelopmental disorders in childhood and affects 3% – 12% of primary school children.^1^ Attention-deficit hyperactivity disorder impairs the social development and academic performance of an affected individual. It also exacts a huge psychosocial and economic burden on the family and the community.^1,2^

It is associated with an increased risk of low self-esteem, poor interpersonal relationships, poor school performance, conduct problems, criminality, substance abuse and sexual promiscuity, which may increase the risk of human immunodeficiency virus (HIV) transmission.^3,4,5^

Attention-deficit hyperactivity disorder is characterised by a pattern of reduced sustained attention and a higher level of activity in children or adolescents than expected for someone of that age and developmental level.^1,6^ Symptoms include behaviours such as excessive talking, difficulty in paying close attention to details, difficulty in organising tasks and activities, fidgeting and an inability to remain settled in appropriate situations amongst others.^6^ According to the Diagnostic and Statistics Manual-5 (DSM-5) criteria, this pattern of behaviours should be present in at least two settings (such as school and home). It must also result in impaired performance in social, educational or work settings.^6^ Three presentations have been described in the DSM-5, and these include the inattention, hyperactivity and the combined.^6^

Attention-deficit hyperactivity disorder is more common in males, but the rate varies amongst the presentations.^7,8^ No single factor has been identified or sufficient to cause the development of ADHD, but a composite interaction of nature and nurture had been shown to be contributory.^7,9^ It is commonly associated with other psychiatric disorders such as oppositional defiant disorder (ODD), conduct disorder (CD), anxiety disorder, mood disorders and psychosis.^8,10^ These associations are possibly because of shared genetic and environmental factors, including maternal illness, obstetric complications, exposure to heavy metals such as lead, substance abuse and head injury.^9,11^

Epidemiological studies had reported varying prevalence rates across cultures and geographical regions of the world, with worldwide prevalence rates of as low as 0.5% to 20%.^12,13^ A prevalence rate of 11% was found amongst children and adolescents in the United States of America^14^ and 0.5% in the United Kingdom.^12^ Data from Africa found prevalence rates of 6% in the Democratic Republic of Congo,^15^ 8.7% in Nigeria^8^ and 23.7% amongst medical students in Kenya.^16^ Anecdotal reports from private and public health facilities had confirmed that ADHD exists in Botswana as elsewhere. Previously, a 25% prevalence of ADHD was reported amongst children from the only tertiary psychiatry hospital,^17^ but the prevalence of ADHD in the community is yet to be ascertained. Furthermore, the youthful population profile in Botswana (Botswana Population Census Atlas 2011) makes this research relevant, as ADHD is predominantly a disorder of youth.^1^ Hence, this study aimed to determine the prevalence of ADHD, the presentations and associated comorbid conditions in a community sample of school children in Gaborone, Botswana. The study also explored the associated factors of ADHD, based on the findings from the parents’ interview. The present study hoped to add to the existing knowledge on the geographical variation and cultural disparities in rates of ADHD and its presentations. It additionally hoped to lay a foundation for further research and mental healthcare formulation for the affected children, who are yet to be identified in the community in Botswana.

## Methods

### Study design and setting

This was a cross-sectional study, which involved parent and teacher survey of primary school pupils, aged 6–12 years. The study was conducted in Gaborone, the capital city of Botswana. Gaborone is the most densely populated city, accounting for approximately 10% of Botswana’s total population (Botswana Population Census Atlas 2011). It is also representative of the reference population regarding the number of primary schools and ethnic distribution.

### Procedure

Two hundred pupils were selected from seven regions in Gaborone to constitute 1400 participants, by using a two-stage sampling technique. Nonetheless, we increased the sample by 30%, anticipating a 30% attrition rate, which was calculated from a pilot study conducted earlier. A sample size of 1820 pupils was thus targeted.

The data collection was carried out in two phases. The teachers were instructed on how to administer the Vanderbilt ADHD Diagnostic Rating Scale-Teacher Version (VADRS-TV) on the pupils whose parents or guardians provided written consent. Only the teachers who have been with the pupils for a minimum of 6 months were interviewed. The research assistants then followed up with parent interviews only for those who met the criteria for ADHD on the teacher version. The parents were instructed on how to rate their children on the parent version of the Vanderbilt ADHD Diagnostic Rating Scale-Parent Version (VADRS-PV). The research assistants helped those unable to read or write by reading out the questions and filling their responses on the empty questionnaires. Only the agreement between the two ratings was regarded as diagnostic of ADHD in this study based on DSM-5 diagnostic criteria of symptoms in two settings.

### Measures

Vanderbilt ADHD Diagnostic Rating Scale^18^ is available in two versions, namely the parent version and the teacher version. These two versions are quite similar and are designed to assess disruptive problems modelled on DSM-IV criteria amongst children aged 6–12 years of age. The teacher version comprises 35 items, whereas the parent version has 47 items. The items represent DSM-IV criteria for ADHD predominantly but include items on the DSM-criteria for the ODD, CD, anxiety and depression. The items are rated on a 4-point scale with responses: ‘never’, ‘occasionally’, ‘often’ and ‘very often’. Only the symptoms that happen ‘often’ or ‘very often’ are regarded as present. The scale also includes eight items on classroom performance, peer relations and organisational skills, which are rated on a 5-point scale, but grouped into three labels, namely problematic, average and above average. Scores of ‘2’ on at least two, or ‘1’ on at least one of the performance questions, that is ‘problematic’ indicates dysfunction. To meet the criteria for the ADHD diagnosis, which is the outcome measure, pupils must have at least six positive responses to either the inattentive or hyperactive nine core symptoms, or both, plus a score that indicates ‘problematic’ in the performance section. These instruments had been used previously in African settings with comparable results.^8,15^ In addition to the questions on the VADRS-PV, some essential socio-demographic questions were asked of the parents. These included age, occupation, educational level, history of any mental illness in the family, history of perinatal complication, maternal illness or use of any substance in pregnancy and the number of children. Questions on the children’s age and gender were also included. The occupation was classified based on the 2008 Botswana Standard Classification of Occupations. It was classified into the upper occupational group (legislators, managers, professionals and large-scale traders), lower occupational group (plant operators, clerks, cleaners, small-scale traders and technicians) and the unemployed. The parent version was translated to Setswana, by using the procedure of iterative back-translation by a panel of bilingual experts.

### Data analysis

Data were analysed by using the Statistical Package for Social Sciences (SPSS) for Windows, version 16.0. The descriptive statistical tools such as frequencies (%), mean and s.d. were used to present the prevalence of ADHD and other demographics. Continuous variables such as age were dichotomised by using the calculated mean as the cut-off. Pearson’s Chi-square tests were used to calculate differences between groups, such as the presence of ADHD, the outcome variable and gender. The relationship between ADHD and comorbid disorders, such as ODD and CD, was also tested by using Chi-square tests. A post hoc analysis was performed on those who were positive by the teachers’ rating. The relationship between identified socio-demographic risk factors and ADHD was explored using Chi-square tests and logistic regression. All tests were two-tailed, and a *p*-value of less than 0.05 was accepted as the level of statistical significance.

### Ethical consideration

Permission to embark on the study was obtained from the Ethics and Research Committee of the University of Botswana (UBR/RES/IRB/BIO/016) and the Ministry of Basic Education (DPRS 7/1/5 XXX (18) PAO-Research). Letters were sent to the schools to inform the teachers and parents or guardians about the study. Their consents to participate were also sought in writing.

## Results

### The demographic profile of the participants

Of the 1820 VADRS-TV distributed to the teachers who consented, 1799 (98.8%) were retrieved after the first phase of the study, and 420 (23.3%) children met the criteria for ADHD. The parents of these 420 participants were subsequently interviewed with VADRS-PV, and 402 parents returned the questionnaires. The questionnaires of those whose parents refused to participate and those with incomplete responses in the variables of interest were excluded. Hence, 1737 (92.4%) questionnaires were finally analysed.

There were slightly more male children than female children (50.9% vs. 49.1%), and the mean age of the participants was 9.53 (s.d. = 1.97) years, with a range from 6 to 12 years.

### Prevalence of attention-deficit hyperactivity disorder and its presentations

The prevalence of ADHD amongst 1737 school-age children according to the teachers’ rating was 23.1% (*n* = 402), by parents’ rating was 13.2% (*n* = 229) and by combined parents’ and teachers’ reports was 12.3% (*n* = 213). The distribution of ADHD presentations was the predominantly hyperactive/impulsive (1.2%, *n* = 20), the predominantly inattentive (7.2%, *n* = 125) and the combined (3.9%, *n* = 68). The male-to-female ratio for the 213 children with ADHD in this study was 2.2:1. Across the presentations, the male : female gender ratios were predominantly inattentive: 2.4:1, hyperactive/impulsive 1.2:1 and combined 3:1. A statistically significant gender difference was observed in the rate of ADHD (χ^2^ = 38.0, *p* < 0.01), the predominantly inattentive presentation (χ^2^ = 20.0, *p* < 0.01) and the combined χ^2^ = 16.4, *p* < 0.01) with male predominance. There was no gender difference in the predominantly hyperactive presentation (χ^2^ = 0.13, *p* = 0.71).

### Comorbid psychiatric disorders

Of the 213 patients who met the criteria for ADHD on the teacher and parent scales, 113 (53.1%) had at least one comorbid psychiatric disorder. The most common psychiatric disorder amongst those who met the criteria was ODD (*n* = 70, 32.9%), especially in the hyperactive-impulsive and combined subgroup. The externalising disorders, ODD (χ^2^ = 39.1, *p* < 0.01) and CD (Fisher’s exact test [FET], *p* < 0.01), were more associated with the hyperactive and the combined presentation, whereas the internalising disorders (χ^2^ = 6.61, *p* = 0.037) more associated with the inattentive presentation.

### Association between the risk factors (socio-demographic and clinical variables), and attention-deficit hyperactivity disorder

A post hoc analysis of the 402 children with parent and teacher ratings was conducted. The age of the pupils, their parents and the family size were dichotomised by using the calculated means for the bivariate analysis.

On bivariate analysis, there was a significant association between male gender and ADHD (Table 1). Other significant findings were a family history of mental illness and a history of perinatal complications (Table 1).

**TABLE 1:** The association between the risk factors and attention-deficit hyperactivity disorder amongst those rated by both parent and teacher (*n* = 402).

Variables	ADHD				Statistics		
	Present		Absent		*χ^2^*	*df*	*p*
	*N*	*%*	*N*	*%*			
**Age group**					1.11	1	0.292
9 years and older	66	49.6	67	50.4			
Below 9 years	117	55.5	94	44.5			
**Gender**					10.6	1	0.001*
Male	142	59.7	96	40.3			
Female	65	42.8	87	57.2			
**Fathers’ age**					0.46	1	0.497
Below 40 years	71	55.0	58	45.0			
40 years and older	64	50.8	62	49.2			
**Mothers’ age**					0.00	1	0.984
34 years and younger	102	53.4	89	46.6			
Older than 34 years	105	53.3	92	46.7			
**No. of children (family size)**					0.61	1	0.434
Four children and less	169	52.8	151	47.2			
More than four children	26	59.1	18	40.9			
**Fathers’ level of education**					3.58	1	0.058
Below secondary	75	48.1	81	51.9			
Secondary school and above†	63	60.0	42	40.0			
**Mothers’ level of education**					2.81	1	0.094
Below secondary	133	50.0	133	50.0			
Secondary school and above	80	58.8	56	41.2			
**Fathers’ occupational status**					0.25	2	0.882
Not employed	9	50.0	9	50.0			
Low skilled employment	97	56.1	76	43.9			
High skilled	32	56.1	25	43.9			
**Mothers’ occupational status**					3.63	2	0.162
Not employed	47	59.5	32	40.5			
Lower occupational group	122	50.6	119	49.4			
Upper occupational group	32	62.7	19	37.3			
**Marital status**					1.05	1	0.305
Separated or not married	184	54.8	152	45.2			
Married and living together	14	45.2	17	54.8			
**A family history of mental illness**					9.38	1	0.002*
Absent	123	46.1	144	53.9			
Present	15	83.3	3	16.7			
**Maternal illness and substance use in pregnancy**					1.89	1	0.168
Absent	176	51.3	167	48.7			
Present	28	62.2	17	37.8			
**Perinatal complications**					6.78	1	0.009†
Absent	154	50.0	154	50.0			
Present	59	53.5	31	34.4			

Risk factors include socio-demographic and clinical variables.

* , Significant *p*-value;

† , Minimum of 12 years of formal education; ADHD, attention-deficit hyperactivity disorder.

In addition to the significant variables on bivariate analysis, fathers’ and mothers’ levels of education, which fell short of significance, were entered into the logistic regression analysis. Only a family history of mental illness (odds ratio [OR] = 6.59, 95% CI: 1.36–32.0) and history of perinatal complications (OR = 2.16, 95% CI: 1.08–4.29) emerged as the independent predictors of ADHD (Table 2).

**TABLE 2:** The regression model of the association between the risk factors† and attention-deficit hyperactivity disorder amongst those rated by both parents and teachers (*n* = 402).

Risk factors	Wald	*p*	OR	95% Confidence interval (CI)	
				Lower	Upper
**Gender**					
Male	2.61	0.106	1.67	0.89	3.11
**Fathers’ level of education**					
Below secondary school	2.89	0.089	0.52	0.24	1.11
**Mothers’ level of education**					
Below secondary school	0.08	0.776	0.89	0.42	1.93
**A family history of mental illness**					
Present	5.47	0.019*	6.59	1.36	32.0
**Perinatal complication**					
Present	4.79	0.029*	2.16	1.08	4.29

* , Significant *p*-value;

† , Risk factors include socio-demographic and clinical variables.

## Discussion

The main finding of this study was the 12.2% prevalence of ADHD based on the agreement between teachers and parents on the Vanderbilt Rating Scale. Other key findings in this study included male predominance in those who met the criteria for ADHD and the independent association between ADHD and clinical variables such as a positive family history of mental illness as well as history of perinatal complications at birth.

The ADHD prevalence rate in this current study is comparable with 11% reported in a study in the USA,^19^ and 13% in some non-western cultures such as Iran^20^ and 12% in Ghana.^21^ The rate is nonetheless higher than 0.5% found in a study in Europe^12^ and other African countries such as Nigeria^8^ and Congo,^15^ where 8.7% and 6%, respectively, were reported. Methodological differences, which include sample size, the nature of the sample and criteria used in the definition of the disorder, have been identified for these disparities.^7,8,22^

In the United Kingdom, more stringent ICD-10 criteria are often preferred to DSM criteria, and doctors working in the United Kingdom are less likely to make a diagnosis of ADHD compared with those in the United States of America.^12,23^ These findings may explain the lower rates reported in the United Kingdom compared with the United States of America. Likewise, the nature of the sample used (hospital record review) and the small sample size may have contributed to the higher rate (25%) of ADHD reported from an earlier study in Botswana.^17^

Even though the variations in rates across nations have mainly been attributed to methodological differences,^7,8,22^ it is noteworthy to find that our rate differs from that of Adewuya and Famuyiwa,^8^ who also used the VADRS and a similar methodology in an African setting. Perhaps, geographical location and local culture similarly play a significant role in the epidemiology, and varying rates of ADHD symptoms reported despite the argument to the contrary. Families, teachers and even clinicians from different cultures around the world vary in their expectations and tolerance of children’s behaviours.^24^ Varying attitudes towards acceptable behaviour and cultural interpretation of symptoms may influence the diagnosis of ADHD, particularly in Africa, where some still attribute mental illness to supernatural affliction. In the current study, the perceptions of parents were different from those of the teachers, which explained the disparity in their ratings. It is possible that parents who spend more time with their children have grown to accept some of the behaviours and, consequently, more tolerable than the teachers who barely know them or spend less time with them. Moreover, it has been suggested that the disparities would be more prominent when a rating scale is used as in this study rather than diagnostic criteria such as DSM, which may be more resistant to cultural influence.^24,25^ Thus, whilst controlling for the influence of different designs on the varying rates of ADHD, the role of cultural differences and geographical location should also not be overlooked, especially in the African setting. Nevertheless, the relatively higher rate from the current study calls for immediate intervention and further research with a diagnostic assessment of children in Botswana.

Notwithstanding that the rate of ADHD in Botswana is slightly higher than reports from most literature,^7^ the pattern of presentation in the current study followed the popular trend, internationally.^8,20,26,27^ The inattentive type was the commonest, followed by the combined presentation. This finding contrasts with a study from Korea,^28^ which reported different patterns of presentation. The influence of culture on the interpretation and appreciation of symptoms of ADHD may also contribute to the variation in the prevalence of ADHD presentations.

Evidence suggests that more than 50% of those who have ADHD have a comorbid psychiatric disorder.^7,10^ Similarly, the present study revealed that 53.1% of those who met the criteria for ADHD according to the rating scales had at least one comorbid psychiatric condition. The most frequently observed comorbid psychiatric disorder was ODD, which occurred in 32.9% of those who met the criteria for ADHD. Furthermore, our study showed that the externalising disorders, ODD and CD, were more associated with the hyperactive and combined presentations, whereas internalising disorders such as anxiety and depression were more associated with the inattentive subgroup. These findings have been replicated by previous literature,^7,29^ which suggested that externalising disorders are more associated with the hyperactive-impulsive presentation of ADHD, whereas the internalising disorders are more associated with the inattentive presentation.

A gender difference was observed in the prevalence of ADHD in the present study. Boys were 2.2 times more likely to have rated positive for ADHD than girls, and this is comparable with the pooled prevalence of 2.45 for boys from a systematic review.^7^ This study did not find any gender difference in the hyperactive presentation as in previous studies with similar designs.^7^ Ramtekkar and colleagues^30^ suggested that the gender difference in the hyperactive/impulsive presentation of ADHD reduces or becomes insignificant as children approach adolescence or adulthood. The fact that more than half (*n* = 13, 59.1%) of those who had hyperactive/impulsive presentations were 10 years and older may have contributed to the insignificant gender difference observed in the present study.

The post hoc analysis revealed a significant association between gender and ADHD in this study. Whilst the initial analysis suggested that gender plays a role in the development of this disorder, the logistic regression analysis proved otherwise. The influence of other variables such as a history of mental illness in the family included in the analysis may have accounted for the significant relationship on bivariate analysis. For instance, gender-specific vulnerability to non-familial risk factors has been shown to mediate the onset of ADHD in males.^31^ Nonetheless, gender roles in the development of ADHD should be further explored.

In the present study, a positive family history of mental illness was associated with ADHD on logistic regression analysis. The fact that ADHD coexists with other psychiatric disorders, as shown in this study, is not a coincidence. Attention-deficit hyperactivity disorder has been genetically linked to various psychiatric disorders. High proportions of psychiatric disorders such as ADHD and CD have been found amongst relatives of individuals with schizophrenia and schizotypal personality disorder.^32^ Authors have also suggested that ADHD symptoms in childhood may be predictors of adult schizophrenia and mood disorders.^32,33^ The co-occurrence of ADHD, schizophrenia, depression, and bipolar disorder suggests that they may not entirely be aetiologically distinct sub-syndromes, but disorders with shared genetic factors. Therefore, it is not surprising that we found six times the risk of ADHD amongst those who have a family history of mental disorder.

Other possible reasons for this association include a lower threshold for behavioural problems in children with ill family members^32^ and greater mental health awareness from previous mental healthcare contact. Further studies may be needed to clarify these. Nevertheless, whilst the present study did not investigate the type of mental disorders, it highlights the need to see childhood disorders such as ADHD as a pointer to adult or other psychiatric disorders. Screening for them in other members of the family or the children with a behavioural disorder may help in early detection and better quality of life.^34^

The complexity observed in the inheritance of ADHD and that it does not follow the Mendelian law of segregation imply an interaction between multiple genes and environmental risk factors as alluded to previously.

In this study, the identified environmental factor associated with ADHD was a history of perinatal complications. This variable was not only found to be associated with ADHD but also suggested that those exposed to perinatal complications have two times the risk of developing ADHD. The current finding is consistent with a previous work, linking perinatal complication with later development of externalising disorders such as ADHD and CD.^35^ Getahun and colleagues^36^ suggested that the adverse effect of ischemic-hypoxic conditions due to birth asphyxia and respiratory distress syndrome on prenatal brain development may lead to functional problems, including ADHD. After controlling for the genetic relatedness of behavioural disorder between parent and child, Wiggs and colleagues^11^ also opined that birth injury and early developmental health hazards might have a significant influence on the ‘neuropsychological processes underpinning’ externalising disorders such as ADHD.

The need to improve on perinatal care and discourage unplanned home delivery cannot be overstressed in this community, especially that an earlier study in Botswana had suggested a link between perinatal complications and childhood mental disorders.^17^

### Limitations and strong points

The present study was conducted in one of the 17 districts in Botswana; thus, generalising the findings to other parts of the country remains hypothetical and should be interpreted with caution. However, the study location has the densest population and is representative of the reference population regarding ethnic distribution.

The present study is limited by the use of parents’ and teachers’ ratings without a clinical diagnostic interview or a direct assessment of the children, to confirm the diagnosis. Nonetheless, the tool used was based on DSM IV-TR criteria and remains valid as DSM-5 still uses the same criteria, except that the symptoms must be present before age 12 years. The use of a sub-sample to explore the risk factors of ADHD also limits our findings.

The strength of the study lies in the use of information by both the teachers and parents to control for over-reporting. Adherence to the DSM-5 diagnostic criteria, one of which emphasises the need for symptoms in two different settings, adds to the strength of this study. Furthermore, it is essential to note that this was the first study to assess the community prevalence of ADHD in Botswana with relatively modest sample size.

## Conclusions

The prevalence rate of ADHD in Botswana is slightly higher than reports from the literature, possibly because of the nature of the instrument used in the present study. The pattern of presentation is nonetheless like the earlier reports, with the predominantly inattentive presentations as the most prevalent and ODD as the commonest comorbid condition. The role of cultural differences in the varying rates of ADHD, especially in the African setting, should be further explored.

Having a positive family history of mental illness and perinatal complications remained the independent predictors of ADHD in the subsample of children with teacher and parent surveys. Hence, mental health screening amongst families of the affected individuals and improved perinatal care should be considered as healthcare priorities in Botswana and other sub-Saharan countries.
